# Supplementary material for: Quantifying benefits of the Danish transfat ban for coronary heart disease mortality 1991–2007: Socioeconomic analysis using the IMPACTsec model
Source: PLoS One. 2022 Aug 17;17(8):e0272744. doi: 10.1371/journal.pone.0272744 (PMC9385018; doi:10.1371/journal.pone.0272744)
Supplement: S1 Appendix — (DOCX) [file pone.0272744.s001.docx]

**Technical Appendix for the Danish IMPACT_SEC_ ITFA Model**

Kirsten Schroll Bjoernsbo*^1^*^¶^, Albert Marni Joensen^2¶^, Torben Joergensen^1,3,4¶^, Soeren Lundbye-Christensen*^5&^*, Anette Bysted*^6^*^¶^, Tue Christensen^6¶^, Sisse Fagt^6¶^, Simon Capewell^7¶^ and Martin O'Flaherty^7¶^

**1** Center for Clinical Research and Prevention, Bispebjerg and Frederiksberg Hospital, Frederiksberg, Denmark

**2** Department of Cardiology, North Denmark Region Hospital, Hjoerring, Denmark

**3** Department of Public Health, Faculty of Health and Medical Sciences, University of Copenhagen, Copenhagen Denmark

**4** Faculty of Medicine, Aalborg University, Aalborg, Denmark

**5** Unit of Clinical Biostatistics, Aalborg University Hospital, Aalborg, Denmark

**6** National Food Institute, Technical University of Denmark, Kgs. Lyngby, Denmark

**7** Department of Public Health and Policy, University of Liverpool, Liverpool, United Kingdom

^¶^These authors contributed equally to this work.

^&^This author also contributed equally to this work.

**List of contents**

**List of abbreviations 3**

**Overview. The IMPACT_SEC_ ITFA model: Introduction, transfat data applied in the model, and sensitivity analysis in relation to cholesterol**

**1. Introduction 4**

**2. ITFA Data – data sources and calculation of transfat content - Beta coefficient 6**

**Table S1. ITFA (E%) levels in 1991 and 2007 by sex and socioeconomic quintiles 7**

**3. Methodological considerations regarding transfat impact on coronary heart disease 8**

**Table S2a-b. Adjusted and unadjusted Sensitivity analyses of ITFA consumption and CHD DPPs 9**

**Reference List 11**

| **List of abbreviations:** | |
| --- | --- |
| **CCHS** | **Copenhagen City Heart Study** |
| **CHD/CVD** | **Coronary heart disease/Cardiovascular diesease** |
| **CPR number**  **Dan-MONICA III** | **Civil personal registry number (a unique personal 10-digit code)**  Multinational MONItoring of trends and determinants in CArdiovascular disease; in 1991 |
| **DANSDA** | Danish National Surveys of Dietary Habits and Physical Activity |
| **GIES** | General Intake Estimation System |
| **HDL / LDL** | High density Cholesterol/Low Density Cholesterol |
| **ITFA** | Industrially produced trans fatty acids |
| **SEC** | **Socio-economic** |
| **SECQ** | **Socio-economic quintile** |
| **TFA** | **Trans Fatty Acids** |
| **TRANSFAIR** | **Transfatty Acids in Foods in Europe: The TRANSFAIR Study** |

**Overview. The IMPACT_SEC_ ITFA model: Introduction, transfat data applied in the model and sensitivity analysis in relation to cholesterol**

**1.** **Introduction**

The Danish IMPACT_SEC_ ITFA model is an extension of the Danish IMPACT_SEC_ model, which accommodates sub-national variation in Coronary Heart Disease (CHD) mortality trends by socioeconomic circumstances. This introduction explains what IMPACT is and how the ITFA model builds upon former IMPACT models as originally presented in Joensen et al’s (2018) introduction to their technical appendix. For further information on methodology and examples of deaths prevented or postponed (DPP) calculations, you are referred to Joensen et al’s technical appendix (<https://doi.org/10.1371/journal.pone.0194793.s001>). This appendix focuses on the extension with industrially produced transfatty acids (ITFA) data and methodological considerations relating to this.

IMPACT is a deterministic, cell-based policy model. It uses epidemiological information to estimate the contributions of population-level risk factor changes (impacting mainly on incidence) and changes in the uptake of evidence-based treatments (impacting mainly on case fatality) between two points in time (the start-year and the end-year).  The primary outcome measure of the model is the deaths prevented or postponed (DPPs).

The starting point for the model is to calculate the ‘target’ number of deaths the model needs to explain. This target number is obtained by using death counts recorded in the official registration system to calculate the difference between the actual observed Coronary Heart Disease (CHD) deaths recorded in the end-year from expected deaths, i.e., simple indirect standardisation: the number that would have occurred in the end-year had the CHD mortality rates remained the same as in the start-year.

The calculation of the modelled estimate of DPPs rests on utilising two well-studied relationships: firstly, that between risk factor change and the relative reduction in CHD mortality; secondly, that between treatment uptake and reductions in case-fatality in patients with a specific form of CHD.

The model applies the relative risk reduction quantified in previous randomised controlled trials and meta-analyses to estimate the mortality reduction attributable to:

a) temporal change in risk factor prevalence (in those without diagnosed CHD) to calculate the DPPs ‘explained’ by specific risk factor trends,

b) net change over the period in the uptake of specific treatments in patients with each specific form of CHD to estimate DPPs ‘explained’ owing to improved 1-year case fatality rates. Great care is taken to avoid double counting the same individuals.

The mortality benefits from the risk factor reduction in the population, and the treatment benefits in patient groups are then summed. Thus, summing uses a cumulative approach (rather than an additive approach), in order to avoid double-counting of benefits in the same individual.

This mortality sum represents the deaths prevented or postponed (DPPs) ‘explained’ by the model.

At the end of the modelling process, the total DPPs ‘explained’ by the model are then compared with the observed fall in deaths (the ‘target’ to be explained).

Model fit is therefore calculated as the difference between the observed deaths and model DPPs, and expressed as the percentage explained. This measures the extent to which the model was successful in explaining the observed change in CHD mortality in the population.

A policy model like IMPACT thus stands in contrast to a typical multivariate regression model.  A typical multivariate regression model represents a statistical approach to describing a single data-set, for instance generated by a single cohort or RCT. In contrast, a policy model such as IMPACT seeks to integrate and synthesise best estimates from a variety of sources to reliably estimate the extent to which a range of factors, acting in combination, explain or predict an outcome.  We did not obtain the parameters for this model by running regressions. Rather, the model incorporates the best coefficients from the largest meta-analysis or randomised controlled trials of the reduction in case fatality attributed to treatment or the independent effect sizes of a unit change in each risk factor on CHD mortality.

***The IMPACT_SEC_ model***

The IMPACT model was extended to accommodate sub-national variation in CHD mortality trends by socioeconomic circumstances (IMPACT_SEC_ model). In the Danish IMPACT_SEC_ model we used information from Statistic Denmark on financial income for the years before the index years (1990 and 2006) for persons alive and living in Denmark 1. of January 1991 and 2007, respectively.  Based on this information the Danish population was divided into socio-economic quintiles (secq) (groups 1-5) as a proxy indicator of socioeconomic circumstances. The distribution into quintiles was based on age- and gender-specified data so we had equally sized quintiles for each age and gender group. In Denmark each citizen has a unique 10 digit personal identification number (civil personal registry number (CPR number)) that can be used to merge information from different registries (e.g. diagnosis, treatment and financial income) on an individual level.  This model examines the effects of changes in treatment uptake and risk factor trends on changes in mortality from coronary heart disease (CHD) among adults in Denmark aged 25 – 84 years, stratified into equal quintiles by population size. The tables included in this supplementary appendix provide details about the sources and methods that were used.

***The IMPACT_SEC_ ITFA model***

To measure the impact of changes in tranfat intake during the period 1991 -2007, the IMPACT_SEC_ model was further extended with data on ITFA intake during the period. In the original model information on risk factor levels in a Danish population across different gender, age, and income groups was obtained from the Dan-MONICA III Study (1991), Copenhagen City Heart Study 3 (1991), and CCHS4 (2003) as well as Health 2006 (2006-2007). To match the time periods, ITFA intake data was obtained from the Dan-MONICA III Study (1991) and the Danish National Surveys of Dietary Habits and Physical Activity (DANSDA, 2005-2008).

**2. ITFA Data – data sources and calculation of transfat - Beta coefficient**

***The Transfat Database***

Until the detrimental health effects of transfatty acids (TFA) were presented by among others Willet et al in the early 1990s interest in TFA intake was limited and TFA data was not part of the Danish Food Composition tables.  It is first in 1995 with the TRANSFAIR Study from 1995 we get an estimate of TFA intake all over Europe (Hulshof et al; 1999) but still, no transfat database. The Center for Clinical Research and Prevention and the DTU, Food Institute, therefore, decided to build a transfat database on the DanishTFA intake from the seventies, where the intake was high and up to today where ITFA has been phased out. While the content of ruminant transfatty acids (RTFA) is relatively constant in foods, mainly depending on fat content in dairy and type of cut in meat products, the content of ITFA is much more complex depending on the type of fat applied, which has varied across foods and time. For the IMPACTsec ITFA model, this involved search for information on ITFA content in margarines, shortenings, and different foods on the Danish market 1991-2007 combined with studies of period-specific recipes for both homemade and industrially produced meals. The DTU Food Institute archives, as well as international papers on former documentation on TFA content, were reviewed and ITFA values were identified and added to the database according to the specific food and time, the data related to (Ministry of Food, Agriculture and Fisheries of Denmark, 2014). In years without analytical results, the ITFA content in foods was estimated based on the results from the years closest to the year in question, e.g. from the assumption of a linear decrease in the ITFA content during the intervening years. This means that the content for each year was reduced proportionally to the years with known levels. In other cases, the ITFA content in foods was assessed constant in a period of time.

For the IMPACTsec ITFA model, ITFA data were calculated from dietary intake registered by 663 men and women (aged 30 to 70 in 1991) from the Dan-MONICA III (7day diary) and 2,792 men and women (aged 25-75 in 2005-2008) participating in the DANSDA (7day diary) using the Gies system applying recipes from the transfat database. In Table S1 ITFA intake (%E) for men and women in the *Dan-MONICA III* (1991) and DANSDA (2005-2008) studies, stratified by socio-economic quintile using income, are described.

**Table S1. ITFA consumption in Danish 25-75 year old men and women in socioeconomic quintiles (based on financial income)**

|  | **Mean ITFA intake as (%E)** | | | |
| --- | --- | --- | --- | --- |
|  | **1991**  ***(Dan-MONICA III Study)*** | | **2005-2008**  **(*DANSDA Survey*)** | |
| **Income levels** | **Men (n=329)** | **Women (n=334)** | **Men (n=1,321)** | **Women (n= 1,471)** |
| **Q1 (Most Affluent)** | **0.94** | **0.87** | **0.11** | **0.10** |
| **Q2** | **1.10** | **1.06** | **0.11** | **0.11** |
| **Q3** | **1.19** | **1.12** | **0.09** | **0.10** |
| **Q4** | **1.13** | **1.13** | **0.09** | **0.10** |
| **Q5 (Most Deprived)** | **0.92** | **1.02** | **0.07** | **0.08** |
| **Average** | **1.06 (0.82)** | **1.04 (0.68)** | **0.09 (0.13)** | **0.10 (0.14)** |

***CHD beta coefficient estimate for transfat***

In Joensen’s (2018) technical appendix (<https://doi.org/10.1371/journal.pone.0194793.s001>) information on β coefficients estimated from multiple regression analyses for the relationship between absolute changes in population mean risk factors and percentage changes in coronary heart disease mortality for men and women, stratified by ag**e** are presented.

The β coefficient estimate for transfat  for the relationship between absolute change in transfat intake and percentage change in CHD mortality for men and women, stratified by age was obtained from the meta-analysis by Mozaffarian et al (2006), using O’Flaherty et al’s (2012) stratification into gender and age-specific mortality reductions.

If 2E% total energy derived from industrial trans fats were completely replaced by monounsaturated and polyunsaturated fats, mortality from CHD would drop by approximately 23%. Thus, if the total energy derived from trans fats were reduced by 0.5E%, the number of CHD deaths would drop by approximately 6%.

**3. Methodological considerations regarding transfat impact on CHD**

***Avoiding double counting the effect of population level changes in cholesterol***

TFA harms operate mainly via effects on LDL and HDL cholesterol. As the original IMPACTsec Model also separately considers reductions in total cholesterol since 1991, some mortality benefits may have been double counted in the ITFA model. However, ITFA reduction decreases CHD risk and mortality via diverse pathways, not just through changes in cholesterol. The potential scale of this potential overestimation can be gauged by rigorous sensitivity analyses.

The model estimate that 2542 DPPs can be explained by changes in cholesterol at population level, excluding the effects of statins.

To avoid double counting, if we subtract the effect of Transfats (1191) from the cholesterol population effect, we obtain, the residual contribution of cholesterol decrease to explain the total mortality CHD fall.

This shows, that even if the mortality benefit were reduced by the entire ITFA 11%, the residual contribution of cholesterol decrease would still explain 12% of the total mortality CHD fall.

***Sensitivity Analysis***

The effect of uncertainty in key parameters were estimated using a montecarlo simulation approach, applying 1000 runs of the full model using the excel add-in Ersatz v1.35 (www.epigear.com).

The key parameters included in the sensitivity analysis included consumption of ITFA using a PERT distribution, link to mortality from coronary heart disease using a log normal distribution, mortality from coronary heart disease and numbers of patients with coronary heart disease using a Poisson distribution , strength of the gradient of socioeconomic class. Full results of the probabilistic sensitivity analysis are presented in Table S2a-b.

| **Table S2a Unadjusted Sensitivity analysis of ITFA consumption and CHD DPPs distributed by sex, age and socioeconomic quintiles**  **in Danes 1991 - 2007** | | | | | | | | | | | | | | | | | |  |
| --- | --- | --- | --- | --- | --- | --- | --- | --- | --- | --- | --- | --- | --- | --- | --- | --- | --- | --- |
|  |  |  |  |  |  |  |  |  |  |  |  |  |  |  |  |  |  |  |
|  | Agreggate DPP | | | Q1 DPP | | | Q2 DPP | | | Q3 DPP | | | Q4 DPP | | | Q5 DPP | | |
|  | median | LCI | UCI | median | LCI | UCI | median | LCI | UCI | median | LCI | UCI | median | LCI | UCI | median | LCI | UCI |
| M 25-34 | **1** | *0* | *3* | **0** | *0* | *0* | **0** | *0* | *0* | **0** | *0* | *1* | **0** | *0* | *1* | **0** | *0* | *1* |
| M 35-44 | **7** | *4* | *10* | **1** | *0* | *1* | **2** | *1* | *2* | **2** | *1* | *3* | **2** | *1* | *3* | **1** | *0* | *1* |
| M 45-54 | **26** | *20* | *34* | **4** | *3* | *5* | **5** | *4* | *7* | **6** | *5* | *8* | **7** | *5* | *8* | **4** | *3* | *6* |
| M 55-64 | **158** | *131* | *188* | **19** | *16* | *23* | **26** | *22* | *32* | **32** | *27* | *38* | **45** | *37* | *53* | **36** | *29* | *42* |
| M 65-74 | **326** | *275* | *381* | **33** | *27* | *38* | **63** | *53* | *73* | **77** | *65* | *90* | **71** | *61* | *84* | **82** | *70* | *96* |
| M 75-84 | **412** | *347* | *478* | **49** | *41* | *57* | **85** | *72* | *98* | **95** | *80* | *110* | **82** | *69* | *95* | **102** | *85* | *117* |
|  |  |  |  |  |  |  |  |  |  |  |  |  |  |  |  |  |  |  |
| F 25-34 | **0** | *0* | *2* | **0** | *0* | *0* | **0** | *0* | *0* | **0** | *0* | *1* | **0** | *0* | *0* | **0** | *0* | *0* |
| F 35-44 | **2** | *0* | *4* | **0** | *0* | *1* | **0** | *0* | *1* | **0** | *0* | *1* | **0** | *0* | *1* | **1** | *0* | *2* |
| F 45-54 | **9** | *6* | *13* | **1** | *0* | *1* | **2** | *1* | *2* | **2** | *1* | *3* | **2** | *1* | *3* | **2** | *2* | *3* |
| F 55-64 | **56** | *44* | *69* | **3** | *2* | *5* | **6** | *4* | *8* | **12** | *9* | *15* | **18** | *14* | *22* | **16** | *13* | *20* |
| F 65-74 | **126** | *104* | *150* | **15** | *12* | *18* | **22** | *18* | *26* | **23** | *19* | *28* | **25** | *20* | *30* | **41** | *35* | *49* |
| F 75-84 | **296** | *249* | *346* | **44** | *37* | *51* | **50** | *42* | *59* | **49** | *41* | *57* | **52** | *44* | *61* | **101** | *86* | *117* |
|  |  |  |  |  |  |  |  |  |  |  |  |  |  |  |  |  |  |  |
| M | **930** | *777* | *1094* | **105** | *86* | *125* | **181** | *151* | *212* | **212** | *177* | *250* | **206** | *173* | *243* | **225** | *189* | *263* |
| W | **490** | *402* | *584* | **63** | *51* | *76* | **80** | *65* | *97* | **86** | *71* | *104* | **98** | *79* | *116* | **162** | *136* | *191* |
| M+W | **1420** | *1179* | *1678* | **168** | *137* | *202* | **261** | *217* | *309* | **299** | *248* | *354* | **304** | *252* | *360* | **388** | *324* | *454* |
|  |  |  |  |  |  |  |  |  |  |  |  |  |  |  |  |  |  |  |
| **Table S2b Adjusted Sensitivity analysis of ITFA consumption and CHD DPPs in Danes distributed by sex, age and socioeconomic quintiles**  **in Danes 1991 - 2007** | | | | | | | | | | | | | | | | | |  |
|  |  |  |  |  |  |  |  |  |  |  |  |  |  |  |  |  |  |  |
|  | Adjusted Agreggate DPP | | | Q1 Adjusted DPP | | | Q2 Adjusted DPP | | | Q3 Adjusted DPP | | | Q4 Adjusted DPP | | | Q5 Adjusted DPP | | |
|  | median | LCI | UCI | median | LCI | UCI | median | LCI | UCI | median | LCI | UCI | median | LCI | UCI | median | LCI | UCI |
| M 25-34 | **1** | *0* | *2* | **0** | *0* | *0* | **0** | *0* | *0* | **0** | *0* | *1* | **0** | *0* | *1* | **0** | *0* | *0* |
| M 35-44 | **6** | *3* | *9* | **1** | *0* | *1* | **1** | *1* | *2* | **2** | *1* | *3* | **2** | *1* | *2* | **1** | *0* | *1* |
| M 45-54 | **24** | *18* | *30* | **4** | *3* | *5* | **5** | *4* | *6* | **5** | *4* | *7* | **6** | *4* | *7* | **4** | *3* | *5* |
| M 55-64 | **134** | *111* | *159* | **16** | *13* | *20* | **22** | *19* | *27* | **27** | *23* | *33* | **38** | *31* | *44* | **30** | *25* | *35* |
| M 65-74 | **270** | *229* | *316* | **27** | *23* | *32* | **52** | *44* | *61* | **64** | *54* | *74* | **59** | *50* | *70* | **68** | *58* | *80* |
| M 75-84 | **357** | *300* | *414* | **43** | *36* | *50* | **74** | *62* | *85* | **81** | *69* | *95* | **71** | *60* | *83* | **89** | *74* | *102* |
|  |  |  |  |  |  |  |  |  |  |  |  |  |  |  |  |  |  |  |
| F 25-34 | **0** | *0* | *1* | **0** | *0* | *0* | **0** | *0* | *0* | **0** | *0* | *0* | **0** | *0* | *0* | **0** | *0* | *0* |
| F 35-44 | **2** | *0* | *4* | **0** | *0* | *1* | **0** | *0* | *1* | **0** | *0* | *1* | **0** | *0* | *1* | **1** | *0* | *1* |
| F 45-54 | **7** | *5* | *11* | **1** | *0* | *1* | **1** | *1* | *2* | **2** | *1* | *3* | **2** | *1* | *2* | **2** | *1* | *3* |
| F 55-64 | **46** | *36* | *57* | **3** | *2* | *4* | **5** | *4* | *6* | **10** | *8* | *12* | **15** | *12* | *18* | **14** | *11* | *17* |
| F 65-74 | **100** | *82* | *119* | **12** | *9* | *15* | **17** | *14* | *21* | **18** | *15* | *22* | **20** | *16* | *23* | **33** | *28* | *39* |
| F 75-84 | **244** | *205* | *284* | **36** | *30* | *43* | **41** | *35* | *49* | **40** | *34* | *47* | **43** | *36* | *50* | **83** | *70* | *96* |
|  |  |  |  |  |  |  |  |  |  |  |  |  |  |  |  |  |  |  |
| M | **792** | *661* | *932* | **91** | *74* | *108* | **154** | *129* | *181* | **180** | *150* | *212* | **175** | *147* | *207* | **192** | *161* | *224* |
| W | **399** | *328* | *477* | **52** | *42* | *63* | **65** | *53* | *79* | **71** | *58* | *85* | **79** | *64* | *94* | **132** | *110* | *155* |
| M+W | **1191** | *989* | *1409* | **143** | *117* | *171* | **220** | *182* | *260* | **250** | *208* | *297* | **254** | *212* | *302* | **324** | *271* | *379* |

**Reference list**

**Gies:** General Intake Estimation System, an inhouse developed software for calculation intake of compounds on basis of dietary registration, recipes and compound content databases.

**Hulshof KF, van Erp-Baart MA, Anttolainen M, Becker W, Church SM, Couet C, Hermann-Kunz E, Kesteloot H, Leth T, Martins I, Moreiras O, Moschandreas J, Pizzoferrato L, Rimestad AH, Thorgeirsdottir H, van Amelsvoort JM, Aro A, Kafatos AG, Lanzmann-Petithory D, van Poppel G (1999).** Intake of fatty acids in western Europe with emphasis on trans fatty acids: the TRANSFAIR Study. Eur J Clin Nutr. 1999 Feb;53(2):143-57. doi: 10.1038/sj.ejcn.1600692. PMID: 10099948.

**Joensen AM, Joergensen T, Lundbye- Christensen S, Johansen MB, Guzman-Castillo M, Bandosz P, et al. (2018) Explaining trends in coronary heart disease mortality in different socioeconomic groups in Denmark 1991-2007 using the IMPACTSEC model. PLoS ONE 13(4): e0194793.** [**https://doi.org/10.1371/journal.pone.0194793**](https://doi.org/10.1371/journal.pone.0194793) **1–13 (2018).**

**Joensen, Albert Marni; Joergensen, Torben; Lundbye-Christensen, Søren; Johansen, Martin Berg; Guzman-Castillo, Maria; Bandosz, Piotr; et al. (2018):** Technical appendix for the Danish IMPACTSEC Model. PLOS ONE. Journal contribution. https://doi.org/10.1371/journal.pone.0194793.s001.

**Ministry of Food, Agriculture and Fisheries of Denmark and the Danish Technical University , National Food Institute (2014).** Danish data on *trans* fatty acids in foods. Ministry of Food, Agriculture and Fisheries of Denmark. <https://www.foedevarestyrelsen.dk/Publikationer/Alle%20publikationer/2014004.pdf> (2014)

**Mozaffarian D, Katan M, Ascheiro A, Stampfer MJ, Willett WC (2006).** Trans fatty acids and cardiovascular disease. N Engl J Med 2006; 354:1601–13 PMID: 16611951GPw13

**O’Flaherty M, Flores-Mateo G, Nnoaham K, Lloyd-William F, Capewell S. (2012).** Potential cardiovascular mortality reductions with stricter food policies in the United Kingdom of Great Britain and Northern Ireland. Bulletin of the World Health Organization 2012; 90:522–531 doi:10.2471/BLT.11.092643 PMID: 22807598.

**Willett WC, Stampfer MJ, Manson JE, Colditz GA, Speizer FE, Rosner BA, Sampson LA, Hennekens CH.** **(1993)** Intake of trans fatty acids and risk of coronary heart disease among women. Lancet. 1993 Mar 6;341(8845):581-5. doi: 10.1016/0140-6736(93)90350-p. PMID: 8094827.
