# Supplementary material for: Quantifying benefits of the Danish transfat ban for coronary heart disease mortality 1991–2007: Socioeconomic analysis using the IMPACTsec model
Source: PLoS One. 2022 Aug 17;17(8):e0272744. doi: 10.1371/journal.pone.0272744 (PMC9385018; doi:10.1371/journal.pone.0272744)
Supplement: Attachment — Submitted filename: Response to reviewers.docx [file pone.0272744.s002.docx]

**Bispebjerg and Frederiksberg Hospital**

Center for Clinical Research and Prevention

Hovedvejen, indgang 5, 1. sal

Nordre Fasanvej 57

2000 Frederiksberg

Denmark

Phone: +45 20 16 91 34

Email: [Kirsten.bjoernsbo@regionh.dk](mailto:Kirsten.bjoernsbo@regionh.dk)

June 30, 2022

**Research Editors, PLOS ONE**

Dear Paolo Piras,

Thank you for the opportunity to submit a revised version of our manuscript PONE-D-22-04093:

**Quantifying benefits of the Danish Transfat Ban for coronary heart disease mortality 1991-2007: socioeconomic analysis using the IMPACTsec model.**

Regarding the journal requirements:

1. The manuscript has been updated according to PLOS ONE’s style requirements.

2. We have included an updated “Competed Interests statement” in our cover letter according to your suggestions.

3. According to Danish law we cannot provide the whole database, but refer to the data in the Technical Appendix. This information has also been added to the cover letter,

4. Full ethics statements including the names for the ethics committee have been included in the revised manuscript:

“*Dan-MONICA III* was approved by the ethics committee of Copenhagen County (KA 90238). The DANSDA surveys are conducted in accordance with the guidelines laid down in the Declaration of Helsinki and are approved by the Danish Data Protection Agency. The Danish National Committee on Health Research Ethics has decided that, according to Danish Law, DANSDA does not require approval. Verbal informed consent was obtained from all participants”.

5. Captions for our supporting information files are now listed in the end of the manuscript

Regarding comments from the reviewer:

Please, find answers to points raised in the table below:

| Comment | Response |
| --- | --- |
| *I can say that I found a lack of clarity in the exposition of statistical methods adopted in the paper. For example the IMPACTsec model is not explained in full and the reader is referred to some references.* | Apologies for the lack of a clarifying paragraph listing the supporting information highlighting the technical appendix.  Our amended manuscript now includes  **Supporting information**  Technical Appendix for the Danish IMPACT_SEC_ ITFA Model including the following tables:  Table S1. ITFA consumption in Danish 25-75 year old men and women in socioeconomic quintiles (based on financial income)  Table S2a Unadjusted Sensitivity analysis of ITFA consumption and CHD DPPs distributed by sex, age, and socioeconomic quintiles in Danes 1991 – 2007  Table S2b Adjusted Sensitivity analysis of ITFA consumption and CHD DPPs in Danes distributed by sex, age, and socioeconomic quintiles in Danes 1991 - 2007 |
| *In my view the paper does not perform a counterfactual evaluation of the Danish ban itself. While the ban has likely reduced trans fat intake/exposure, there are general trends and other confounding factors that may have contributed to trans fat declines and associated health improvements over the period. This would require a control group or natural experiment approach.* | Thank you. We agree that one would ideally want a control group.  However, that is challenging when considering comparisons between countries.  Our amended manuscript now     a) highlights the substantially larger transfat reduction achieved in Denmark compared with other countries in the Limitations paragraph of the manuscript Discussion.  *“Compared to other strategies, the Danish package of voluntary changes by the industry and legislation provides the largest observed reduction in total transfat intake in a population over the period 1976 to 2005 (4.5 g/day) according to Hyseni et.al’s systematic review from 2021 [18]”.*     b) Acknowledges this issue in the Limitations paragraph of the manuscript Discussion.  "*This analysis does not provide a counterfactual evaluation of the Danish ban itself. That would ideally require a control group or natural experiment approach to better account for general trends and other potential confounding factors.  However, the ban likely explains much of the decrease in transfat intake observed over the period, and hence approximately one tenth of the overall fall in cardiovascular disease."* |

Yours sincerely,

Kirsten Schroll Bjørnsbo, PhD, MSc

Albert Marni Joensen

Torben Joergensen

Soeren Lundbye-Christensen

Anette Bysted

Tue Christensen

Sisse Fagt

Simon Capewell

Martin O’Flaherty
